# Screening of Inherited Retinal Disease Patients in a Low‐Resource Setting Using an Augmented Next‐Generation Sequencing Panel

**DOI:** 10.1002/mgg3.70046

**Published:** 2024-12-16

**Authors:** Nicole Midgley, George Rebello, Lara K. Holtes, Raj Ramesar, Lisa Roberts

**Affiliations:** ^1^ University of Cape Town/MRC Precision and Genomic Medicine Research Unit, Division of Human Genetics, Department of Pathology, Institute of Infectious Disease and Molecular Medicine, Faculty of Health Sciences University of Cape Town Cape Town South Africa

**Keywords:** deep intronic variants, inherited retinal disease, targeted next‐generation sequencing

## Abstract

**Background:**

Inherited retinal diseases (IRDs) are a clinically and genetically heterogeneous group of disorders affecting millions worldwide. Despite the widespread adoption of next‐generation sequencing (NGS) panels, there remains a critical gap in the genetically diverse and understudied African populations.

**Methods:**

One hundred and thirty‐five South African patients affected by various IRDs underwent NGS using a custom‐targeted panel sequencing over 100 known genes. The panel was supplemented by *in silico* screening for a *MAK*‐Alu insertion and screening of seven functionally established deep intronic variants.

**Results:**

Through our combined screening strategy, we obtained a probable genetic diagnosis for 56% of the cohort. We identified 83 unique variants in 29 IRD genes underlying the disease, including 16 putative novel variants. Molecular findings prompted recommendations for clinical re‐examination in ten patients. Resolution rates varied across clinical classifications and population groups.

**Conclusions:**

This study reports the first use of a targeted NGS panel for IRDs in southern Africa, demonstrating a cost‐effective, customisable approach that optimises both diagnostic yield and resource efficiency, making it a valuable tool for IRD molecular characterisation in resource‐limited settings. Augmenting the panel by screening for variants relevant to South African patients allowed us to achieve a resolution rate in line with international studies. Our study underscores the importance of investigating diverse populations to bridge disparities in genomic research and improve diagnostic outcomes for underrepresented population groups.

## Graphical abstract

A gene panel, supplemented by deep intronic variant screening, genetically resolved 56% of South African inherited retinal disease patients, including 49% of indigenous Africans. This approach was successfully applied in a low‐resource setting, and contributes knowledge from the understudied, genetically diverse African population.

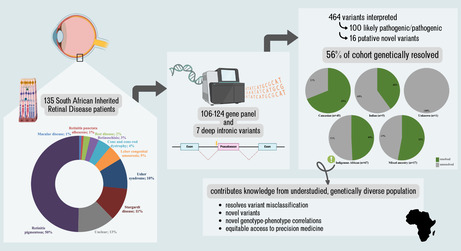

## 1 Introduction

Inherited retinal diseases (IRDs) comprise a highly variable group of hereditary disorders characterised by vision loss due to the degeneration of photoreceptors (rods and cones) and retinal pigment epithelium (RPE) cells. IRDs are a key contributor to the aetiology of vision loss worldwide and have an estimated combined incidence of 1:3500 individuals (Nishiguchi and Rivolta 2012). IRDs can be classified according to: (1) the primarily affected photoreceptor cell (rod‐specific, cone‐specific or combined rod and cone involvement), (2) the disease progression (progressive or stationary) or (3) the presence or absence of extraocular systemic manifestations (syndromic or non‐syndromic) (Berger, Kloeckener‐Gruissem, and Neidhardt 2010).

IRDs exhibit significant phenotypic variability, vast genetic heterogeneity (both locus and allelic) and extensive clinical variation (Cremers et al. 2018). To date, causal variants have been identified in over 300 genes, inherited in an autosomal dominant, autosomal recessive, X‐linked or mitochondrial fashion (RetNet https://retnet.org/). Due to the complexity associated with IRDs, obtaining a molecular diagnosis is paramount. The intersecting clinical phenotypes may confound the clinical diagnosis; thus, molecular testing is required to refine or confirm the diagnosis. Furthermore, a genetic diagnosis may aid in patient management, clarify the prognosis, allow familial testing for risk prediction, impact family planning and determine eligibility for gene‐based clinical trials (Stone et al. 2017; Tayebi et al. 2019; Wang et al. 2013).

The advent of next‐generation sequencing (NGS) has improved molecular diagnostic rates and reduced costs for IRDs by permitting the simultaneous screening of numerous genes in a single assay (Audo et al. 2012; Consugar et al. 2015; Eisenberger et al. 2013; Neveling et al. 2012; Song et al. 2011; Wang et al. 2014). NGS panels can be designed to sequence known disease‐associated genes and are comparatively more time‐efficient and cost‐effective than whole‐exome sequencing (WES) and whole‐genome sequencing (WGS) (Rehm et al. 2013; Stone et al. 2017). Although WES and WGS capture additional genetic information, they necessitate greater computing and analytical resources. Furthermore, focusing exclusively on known IRD genes permits maximal coverage of target genes and provides an ethical advantage by minimising the risk of incidental findings (Dockery et al. 2021). Stone et al. (2017) reported that using a clinically directed, tiered‐testing strategy with panel data for IRDs can: (i) increase the sensitivity by 6.1% and (ii) reduce the average cost per patient by 17.7% in comparison to WES. The authors suggested that the false genotype rate (FGR) can be reduced by using clinical information to narrow the pre‐test hypothesis to the smallest number of genes possible (Stone et al. 2017). Several IRD panels are available internationally, ranging from broad (for general eye disorders) to highly specific (for a specific IRD subtype), with an average detection rate of 40%–70% (Dockery et al. 2021; Neveling et al. 2012; Stone et al. 2017).

Despite the enhanced diagnostic rates achieved through NGS, 30%–40% of IRD patients remain genetically unresolved (Bravo‐Gil et al. 2016; Gonzalez‐Del Pozo et al. 2018; Perez‐Carro et al. 2016). A significant proportion of this missing heritability has recently been attributed to deep intronic variants (DIVs), which are located beyond the splicing motifs of exon‐intron boundaries, typically over 100 base pairs (bp) into the intron (Vaz‐Drago, Custodio, and Carmo‐Fonseca 2017). DIVs can result in disease due to aberrant splicing (Cho et al. 2020; Popp and Maquat 2013; Vaz‐Drago, Custodio, and Carmo‐Fonseca 2017; Weisschuh, Buena‐Atienza, and Wissinger 2021) and have been identified, with functional validation, in multiple IRD genes including *CEP290* (den Hollander et al. 2006), *USH2A* (Liquori et al. 2016), *CHM* (Carss et al. 2017; Garanto et al. 2018), *PRPF31* (Rio Frio et al. 2009), *OFD1* (Webb et al. 2012) and *ABCA4* (Albert et al. 2018; Zernant et al. 2018).

It is well known that indigenous African populations, although the most genetically diverse, are underrepresented in genomics (Bentley, Callier, and Rotimi 2020; Lumaka et al. 2022). This understudied population may provide novel insights into IRD pathogenicity; we have previously shown through WES in indigenous African IRD patients that ~70% of identified causal variants were novel; however, the majority occurred in known IRD genes (Roberts et al. 2016; Roberts 2017). Here, we present the first‐tiered next‐generation screening strategy for IRD patients in a low‐resource setting.

## 2 Methods

### 2.1 Editorial Policies and Ethical Considerations

This study was approved by the Human Research Ethics Committee (HREC) of the University of Cape Town (UCT) Faculty of Health Sciences (HREC reference numbers: 226/2010 and 576/2019). Informed consent was obtained from each participant at recruitment according to the tenets of the Declaration of Helsinki (World Medical Association 2013).

### 2.2 Cohort

The UCT IRD registry contains biological material, clinical information and demographic data from over 3500 South African IRD families, who have been recruited over a span of 30 years. For this study, a cohort of 135 genetically unresolved, unrelated South African patients, affected by various IRDs, was selected from the UCT IRD registry. Patients were eligible for inclusion if a confirmation of diagnosis (COD) was obtained from an ophthalmologist and, ideally, if familial DNA samples were available. No additional factors, such as specific IRD or ethnolinguistic group, were considered, ensuring the establishment of an unbiased cohort. Clinical diagnoses were established by qualified ophthalmologists, through clinical examination and various diagnostic methods, including electroretinography, colour fundus photography, fluorescein angiography, visual acuity assessment, visual field assessment and optical coherence tomography.

### 2.3 DNA Sample Preparation

Genomic DNA was extracted from blood or saliva samples using a modified version of the salting‐out method (Miller, Dykes, and Polesky 1988), or the Oragene saliva kit (DNA Genotek, Ottawa, Ontario, Canada), respectively, according to the manufacturer's instructions. The DNA was resuspended in 1X Tris‐EDTA buffer and stored at −4°C or −20°C until use. DNA concentration, quality and purity were assessed using the Nanodrop ND1000 Spectrophotometer (Thermo Fisher Scientific, Waltham, Massachusetts, United States), the TaqMan RNase P assay (Thermo Fisher Scientific), agarose gel electrophoresis and the Qubit dsDNA HS Assay Kit (Thermo Fisher Scientific).

### 2.4 Custom NGS Panel Design

A list of genes curated from RetNet (RetNet https://retnet.org/) and Online Mendelian Inheritance in Man (OMIM https://www.omim.org/) (McKusick‐Nathans Institute of Genetic Medicine) was utilised with the Ion AmpliSeq Designer Software (Thermo Fisher Scientific) to develop a custom Ion AmpliSeq On‐Demand Panel (Thermo Fisher Scientific). ‘On‐Demand’ genes are specifically designed to target the gene's coding DNA sequence, including a 5 or 25 bp intronic flanking sequence on both the 5′ and 3′ ends, and have been wet‐bench validated and optimised for high performance by Thermo Fisher Scientific. The panel design was updated over the course of the study as additional genes became available (Table 1). The genes included on the panel were associated with various IRDs, including Best disease (BEST), cone or cone‐rod dystrophy(CRD), Leber Congenital Amaurosis (LCA), macular degeneration (MD), retinitis pigmentosa (RP), Usher syndrome (USH), retinitis punctata albescens (RPA), retinoschisis (RS) and retinoblastoma (Table 2) (Table S1).

**TABLE 1:** Description of panel versions.

Panel version	Number of genes	Number of amplicons	Predicted coverage (%)	Target size (kb)	Number of patients screened
1	106	2904	99	548.69	24
2	116	3096	99	583.94	48
3	124	3302	99	624.18	63

**TABLE 2:** Gene list with corresponding panel version of first inclusion.

Gene	Transcript	Panel version	Gene	Transcript	Panel version	Gene	Transcript	Panel version
*ABCA4*	NM_000350.3	1	*FAM161A*	NM_001201543.2	1	*PRCD*	NM_001077620.3	2
*ABHD12*	NM_015600.5	3	*FSCN2*	NM_001077182.3	1	*PRDM13*	NM_021620.4	1
*ADAM9*	NM_003816.3	1	*GNAT2*	NM_005272.5	1	*PROM1*	NM_006017.3	1
*ADGRA3*	NM_145290.4	3	*GUCA1A*	NM_000409.5	1	*PRPF3*	NM_004698.4	1
*ADGRV1*	NM_032119.4	1	*GUCA1B*	NM_002098.6	1	*PRPF31*	NM_015629.4	1
*ADIPOR1*	NM_015999.6	1	*GUCY2D*	NM_000180.4	1	*PRPF6*	NM_012469.4	3
*AHR*	NM_001621.5	2	*HARS1*	NM_002109.6	1	*PRPF8*	NM_006445.4	1
*AIPL1*	NM_014336.5	1	*HGSNAT*	NM_152419.3	1	*PRPH2*	NM_000322.5	1
*ARL6*	NM_177976.3	1	*HK1*	NM_033497.2	1	*RAX2*	NM_032753.4	2
*ATF6*	NM_007348.4	1	*HMCN1*	NM_031935.3	1	*RB1*	NM_000321.3	2
*BBS1*	NM_024649.5	1	*IDH3B*	NM_006899.5	1	*RBP3*	NM_002900.3	1
*BBS2*	NM_031885.5	1	*IFT140*	NM_014714.4	1	*RD3*	NM_001164688.2	1
*BEST1*	NM_001139443.2	1	*IFT172*	NM_015662.3	1	*RDH12*	NM_152443.3	1
*C1QTNF5*	NM_015645.5	1	*IMPDH1*	NM_000883.4	1	*RDH5*	NM_001199771.2	1
*CA4*	NM_000717.5	1	*IMPG2*	NM_016247.4	1	*RGR*	NM_002921.3	1
*CABP4*	NM_145200.5	1	*IQCB1*	NM_001023570.4	1	*RHO*	NM_000539.3	1
*CACNA1F*	NM_001256789.3	1	*KCNJ13*	NM_002242.4	1	*RIMS1*	NM_014989.5	1
*CACNA2D4*	NM_172364.5	1	*KCNV2*	NM_133497.4	1	*RLBP1*	NM_000326.5	1
*CDH23*	NM_022124.6	1	*KIAA1549*	NM_001164665.2	3	*ROM1*	NM_000327.4	1
*CDH3*	NM_001793.6	2	*KLHL7*	NM_001031710.3	1	*RP1*	NM_006269.2	1
*CDHR1*	NM_033100.4	1	*LCA5*	NM_001122769.3	1	*RP2*	NM_006915.3	1
*CEP290*	NM_025114.4	1	*LRAT*	NM_004744.5	1	*RPE65*	NM_000329.3	1
*CERKL*	NM_201548.5	1	*MAK*	NM_001242957.3	1	*RPGRIP1*	NM_020366.4	1
*CFAP410*	NM_001271441.2	1	*MERTK*	NM_006343.3	2	*RS1*	NM_000330.4	2
*CFAP418*	NM_177965.4	1	*MFSD8*	NM_152778.3	1	*SAG*	NM_000541.5	1
*CFH*	NM_000186.4	1	*MVK*	NM_001114185.3	1	*SEMA4A*	NM_001193300.2	1
*CIB2*	NM_006383.4	1	*MYO7A*	NM_000260.4	1	*SNRNP200*	NM_014014.5	1
*CLRN1*	NR_046380.3	1	*NEUROD1*	NM_002500.5	1	*SPATA7*	NM_018418.5	1
*CNGA1*	NM_001142564.2	1	*NMNAT1*	NM_022787.4	1	*TIMP3*	NM_000362.5	1
*CNGA3*	NM_001298.3	1	*NR2E3*	NM_016346.4	1	*TOPORS*	NM_005802.5	1
*CNGB1*	NM_001297.5	1	*NRL*	NM_006177.5	2	*TRNT1*	NM_182916.3	1
*CNGB3*	NM_019098.5	1	*OFD1*	NM_003611.3	1	*TTC8*	NM_144596.4	1
*CNNM4*	NM_020184.4	1	*OTX2*	NM_001270525.2	1	*TULP1*	NM_003322.6	1
*CRB1*	NM_201253.3	1	*PCARE*	NM_001029883.3	1	*UNC119*	NM_005148.4	1
*CRX*	NM_000554.6	1	*PCDH15*	NM_001142763.2	1	*USH1C*	NM_153676.4	2
*CYP4V2*	NM_207352.4	1	*PDE6A*	NM_001142763.2	1	*USH1G*	NM_173477.5	1
*DHDDS*	NM_024887.3	1	*PDE6B*	NM_000283.4	1	*USH2A*	NM_206933.4	1
*DTHD1*	NM_001170700.3	3	*PDE6C*	NM_006204.4	1	*WHRN*	NM_015404.4	1
*EFEMP1*	NM_001039348.3	1	*PDE6G*	NM_002602.4	1	*ZNF408*	NM_001184751.2	3
*ELOVL4*	NM_022726.4	1	*PDE6H*	NM_006205.3	3	*ZNF513*	NM_144631.6	2
*EMC1*	NM_015047.3	3	*PITPNM3*	NM_031220.4	1			
*EYS*	NM_001142800.2	1	*POMGNT1*	NM_001243766.1	1			

### 2.5 NGS and Ion Torrent Software Analysis

Ion AmpliSeq DNA libraries were prepared using either the AmpliSeq Library Kit Plus (Thermo Fisher Scientific) or the Ion AmpliSeq Kit for Chef DL8 (Thermo Fisher Scientific) and quantified using the Ion Library TaqMan Quantitation Kit (Thermo Fisher Scientific) on the CFX96 Real‐Time System (Bio‐Rad Laboratories, Hercules, California, United States), according to the manufacturer's protocols. Templating and sequencing were performed using the Ion Chef Kit and instrument (Thermo Fisher Scientific). This process produced enriched ion sphere particles (ISPs), which were loaded onto either a P1 Chip v3 and sequenced using the Ion Proton Sequencer (Thermo Fisher Scientific), or onto a Ion 540 Chip and sequenced using the Ion S5 Sequencing (Thermo Fisher Scientific).

Sequencing data were aligned to the hg19 human reference genome using the Torrent Mapping Alignment Program. Subsequently, BAM files were uploaded onto the Ion Reporter Software (Thermo Fisher Scientific) and processed through a custom workflow tailored for each panel version. Automated variant calling and annotation generated a variant call file (VCF) and quality control metrics.

The overall performance of each sequencing run was comprehensively evaluated. Key metrics assessed included ISP density, polyclonal percentage, total number of reads generated per run and sample, percentage of low‐quality reads, read length distribution, percentage of aligned versus unaligned reads, alignment quality and target base coverage. Any amplicons with no coverage in two or more samples were further investigated using the Ion AmpliSeq Designer Software (Thermo Fisher Scientific) to ascertain the predicted coverage of these amplicons.

Prior screening using other methods had been performed for 25 patients of the cohort, which identified genetic variants but left these patients genetically unresolved (i.e., benign, likely benign or variants of uncertain significance were identified, or a single heterozygous pathogenic or likely pathogenic variant was identified in an autosomal recessive gene). These screening results were compared to the variants called in the NGS data to further evaluate the sensitivity of the panel.

### 2.6 Variant Prioritisation and Classification

**FIGURE 1:**
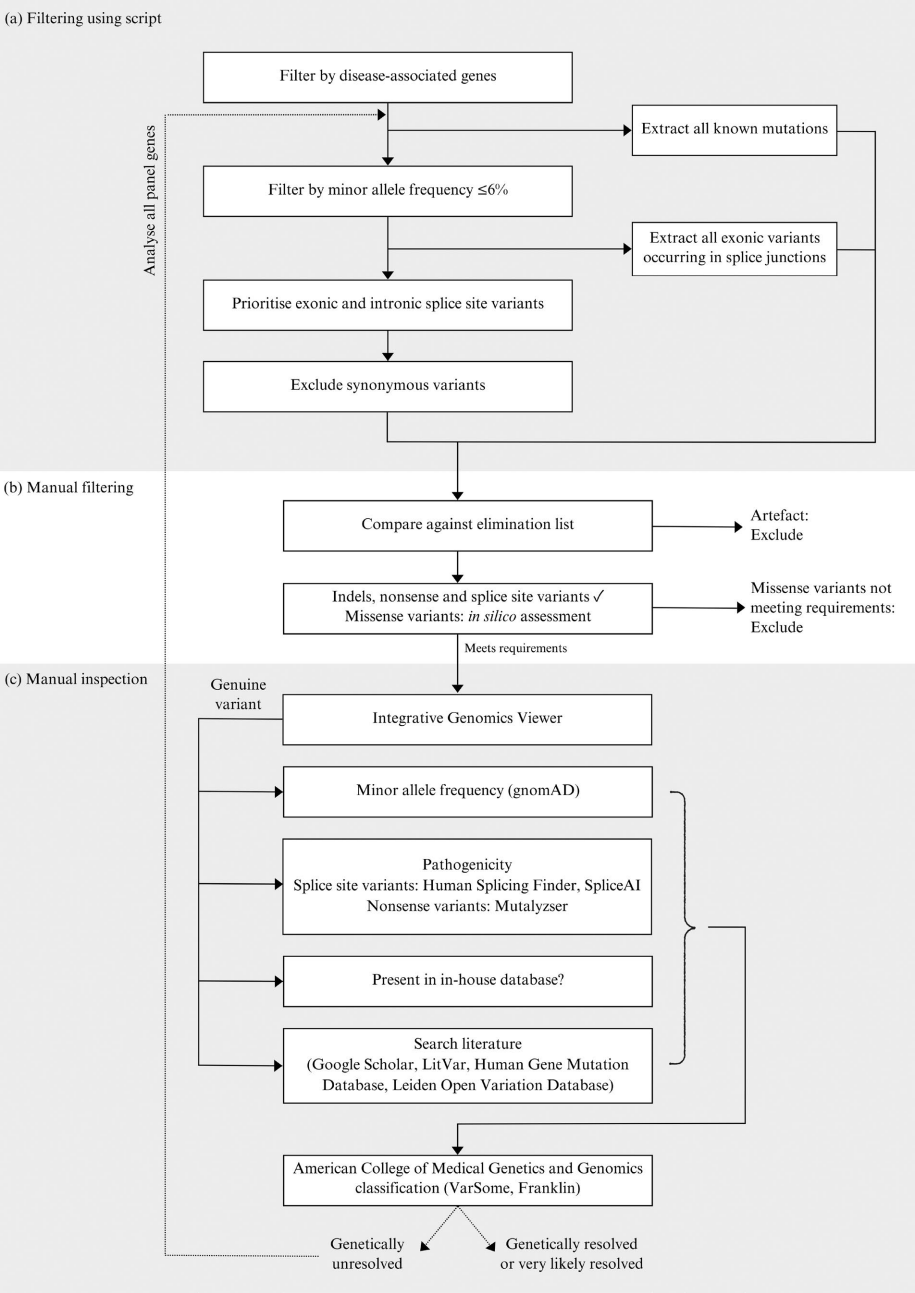
Overview of the analysis pipeline utilised for variant prioritisation and classification in the next‐generation sequencing data.

Each VCF was filtered to identify potential disease‐causing variants (Figure 1). Initial filtering was performed using a Perl script designed to:

1. Select the subset of genes known to be associated with the patient's specific IRD.
2. Flag known disease‐causing variants that have appeared in ≥ 2 families of the UCT IRD registry.
3. Filter by a relatively lenient global minor allele frequency (MAF) cut‐off of 6%, based on the frequency of the most common pathogenic variants in the autosomal recessive IRD gene, *ABCA4* (Stone et al. 2017).
4. Flag all exonic variants occurring in splice junctions (i.e., two bases at both the 5′ and 3′ end of the exon) by utilising a curated list of the chromosomal positions of exon/intron boundaries for the genes in the panel, created using the hg19 human reference genome. It was essential to develop this exon boundary list, as the Ion Reporter Software only flagged canonical intronic variants as potential splice site variants. Without our additional exon boundary analysis, missense or synonymous variants occurring within the first or last two bases of an exon would be erroneously filtered out.
5. Exclude synonymous variants.

Splice site variants, indels and nonsense variants were automatically prioritised for further manual inspection, whereas missense variants required a pathogenic prediction by at least three of the five pathogenicity annotations included in the Ion Reporter Software, i.e. SIFT, Poly‐Phen2, Grantham, FATHMM and PhyloP.

Manual inspection analysis first included an assessment with Integrative Genomics Viewer (IGV) (Robinson et al. 2011) to verify that the variant was not an artefact, through the inspection of read depth, strand bias and occurrences within the cohort. Based on this assessment, an elimination list was curated to exclude variants identified as artefacts (Table S2). For genuine variants, the MAF was examined in all gnomAD populations (Karczewski et al. 2020) to assess the rarity of the variant. All identified canonical splice site variants were analysed using Human Splicing Finder (HSF) (Desmet et al. 2009) to evaluate potential splicing disruption by assessing splice site strength and motifs, and SpliceAI (Jaganathan et al. 2019) was employed to further evaluate splicing impact, with a SpliceAI score > 0.1 used as evidence of potential pathogenicity. All nonsense variants were assessed using a Mutalyzer (Lefter et al. 2021) to evaluate the premature termination of protein translation, including inspecting the length and location of the predicted protein truncation. The variants were searched across published literature using Google Scholar, LitVar (Allot et al. 2018), the Human Gene Mutation Database (HGMD) (Stenson et al. 2017) and the Leiden Open Variation Database (LOVD) (Fokkema et al. 2011), and an in‐house database. Finally, variants were classified according to the American College of Medical Genetics and Genomics (ACMG) guidelines, aided by the online tools VarSome and Franklin (https://franklin.genoox.com; Kopanos et al. 2019; Richards et al. 2015). Each individual case was carefully evaluated, with a thorough inspection of the online tool outputs, and the addition of relevant information on the variant and patient (e.g., zygosity, familial co‐segregation of the variant with phenotype, frequency in an internal database and clinical phenotype). For all genetically unresolved samples at this stage, this filtering process was expanded to investigate all genes included in the panel. Patients with a probable genetic diagnosis were either classified as ‘genetically resolved’ or ‘very likely resolved’. ‘Genetically resolved’ classification required either: (i) a single heterozygous pathogenic/likely pathogenic variant in an autosomal dominant or X‐linked gene; (ii) a single homozygous pathogenic/likely pathogenic variant in an autosomal recessive gene; or (iii) two heterozygous pathogenic/likely pathogenic variants in an autosomal recessive gene where familial testing confirmed the *trans* configuration of mutations. If no familial samples were available for phase confirmation, the patient was classified as ‘very likely resolved’.

All molecularly resolving variants were verified by Sanger sequencing and, where possible, screened for in familial samples for disease co‐segregation and/or phase confirmation. Primers were designed utilising various tools, including Ensembl (Harrison et al. 2024), Primer3Plus (Untergasser et al. 2012), National Center for Biotechnology Information (NCBI) Primer‐BLAST (Ye et al. 2012) and UCSC *In Silico* PCR (Kent et al. 2002) (Table S3). PCRs were conducted on a SimpliAmp Thermal Cycler (Thermo Fisher Scientific) according to the optimised conditions for each primer set detailed in Table S3. Sanger sequencing was performed on an Applied Biosystems 2720 Thermal Cycler (Thermo Fisher Scientific) and subjected to capillary electrophoresis with an ABI PRISM 3130xl Genetic Analyser (Thermo Fisher Scientific). The sequences were captured using Foundation Data Collection Software v3.1.1 (Thermo Fisher Scientific) and aligned to the reference sequence using BioEdit v7.1 (Hall 1999).

### 2.7 *MAK* ‐Alu Screening

A 353 bp Alu repeat insertion in the *MAK* gene, associated with RP, has been reported as a founder variant in patients of Jewish ancestry (Bujakowska et al. 2015; Venturini et al. 2015). However, this variant is not detected by standard NGS pipelines. Therefore, a Perl script was adapted from Bujakowska et al. (2015) to screen for the *MAK*‐Alu insertion in FASTQ files. The script exploits the 23 bp sequence distinguishing wild‐type and mutant *MAK* to count the number of forward and reverse reads containing the wild‐type (5′‐AGGAAAAAAGATTCTCCATTTCG‐3′) versus mutant sequence (5′‐GAAAAAAGGAGGCCGGGCGCGGT‐3′).

All 135 FASTQ files generated from the targeted NGS were screened for the *MAK*‐Alu insertion using the developed script. Additionally, two control files, available from https://github.com/MEEIBioinformaticsCenter/grepsearch, were screened using the designed script. Analysis of the two control files produced the expected results, that is the testA file contained 100% mutant reads (14 mutant and zero wild‐type reads) whereas the testB file contained 36% mutant reads (13 mutant and 23 wild‐type reads).

### 2.8 Deep Intronic Variant Screening

A literature search was conducted to identify published disease‐causing DIVs underlying IRDs and were prioritised based on the following criteria: (i) functional evidence from RNA assays demonstrated a deleterious effect on splicing and (ii) reported in multiple international probands. Variants previously detected in South African IRD patients were of particular interest.

Seven DIVs in three IRD‐associated genes were selected for supplemental screening (Table 3) by either restriction fragment length polymorphism (RFLP) analysis or Sanger sequencing. Primer design, PCR and Sanger sequencing were conducted as described above (Table S3).

**TABLE 3:** Deep intronic variants selected for supplemental screening.

Gene and transcript	Coding	Protein	rsID	mRNA consequence	Number of entries in the Leiden Open Variation Database^a^	Number of South African probands^a^
*ABCA4* NM_000350.3	c.769‐784C>T	p.[=,Leu257Aspfs*3]	rs144695319	Pseudoexon inclusion (Sangermano et al. 2019)	8	1
*ABCA4* NM_000350.3	c.859‐640A>G	p.Phe287Tyrfs*69	No rsID	Pseudoexon inclusion (Khan et al. 2020)	0	2
*ABCA4* NM_000350.3	c.4253+43G>A	p.[=,Ile1377Hisfs*3]	rs61754045	Exon skipping (Sangermano et al. 2019)	58	1
*ABCA4* NM_000350.3	c.4539+2001G>A	p.[=,Arg1514Leufs*36]	rs1457937638	Pseudoexon inclusion (Albert et al. 2018)	41	24
*ABCA4* NM_000350.3	c.5196+1056A>G	p.Met1733Valfs*2	rs886044749	Pseudoexon inclusion (Braun et al. 2013)	8	1
*CEP290* NM_025114.4	c.2991+1655A>G	p.Cys998*	rs281865192	Pseudoexon inclusion (Sangermano et al. 2019)	8	2
*USH2A* NM_206933.4	c.7595‐2144A>G	p.Lys2532Thrfs*56	rs786200928	Pseudoexon inclusion (Sangermano et al. 2019)	39	0

*Note:* * indicate a translation termination (stop) codon, in accordance with Human Genome Variation Society nomenclature.

^a^ As of April 2020, prior to implementation of the current study.

## 3 Results

### 3.1 Cohort

**FIGURE 2:**
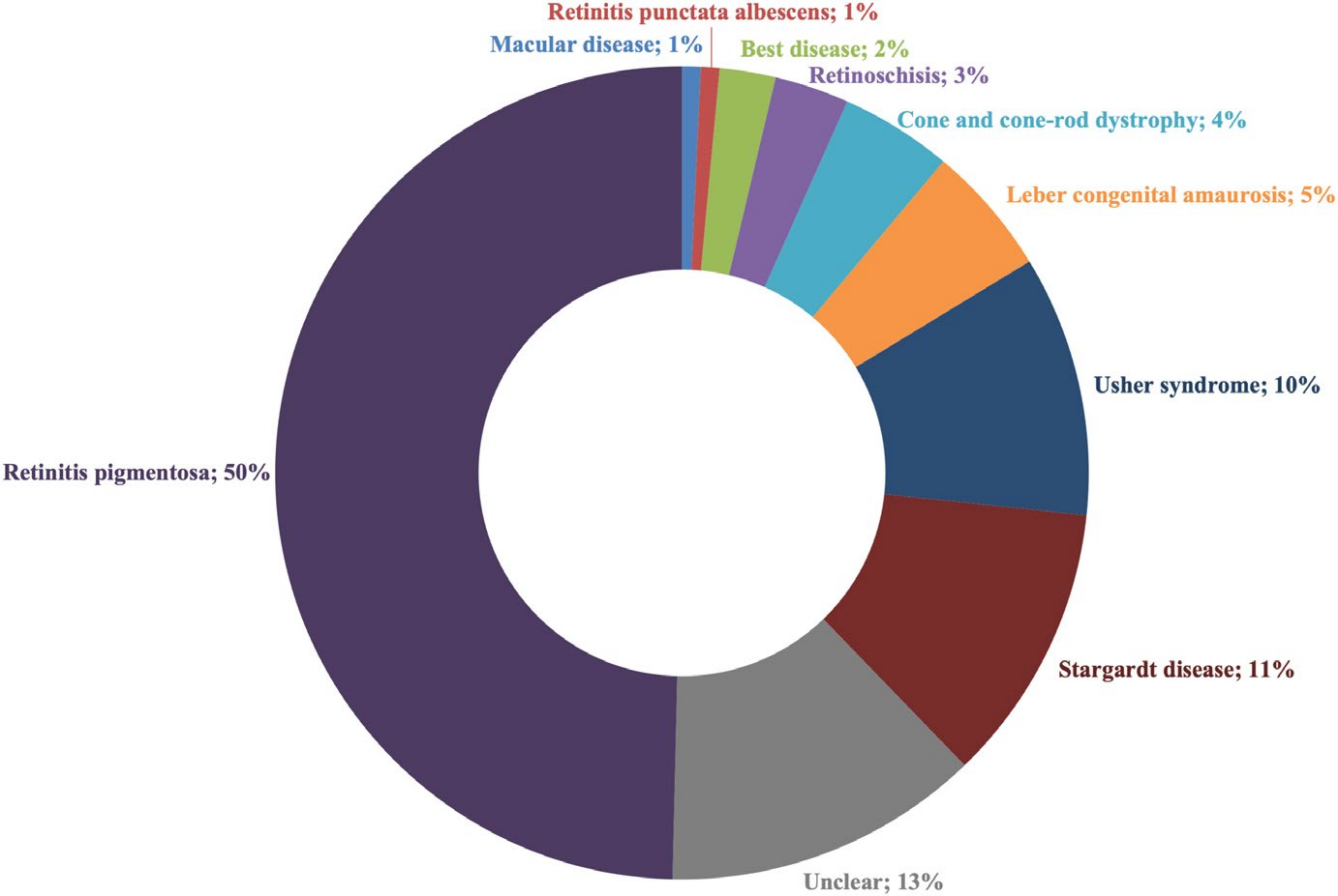
Cohort breakdown by clinical classification.

**FIGURE 3:**
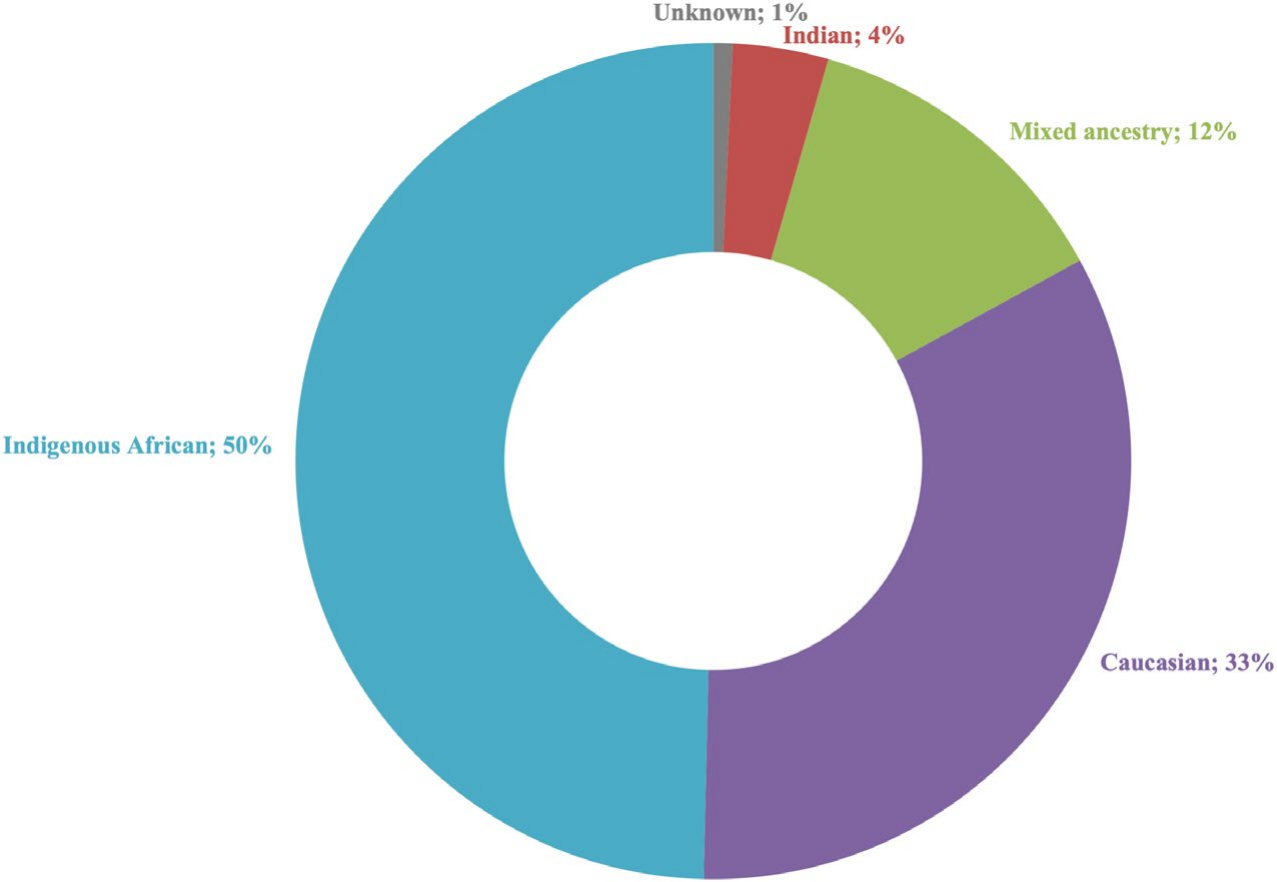
Cohort breakdown by population group.

The patient cohort (*n* = 135) comprised 56 females and 79 males exhibiting various IRD phenotypes, including BEST (2%), CRD (4%), LCA (5%), RP (50%), RPA (1%), RS (3%), STGD (11%) and USH (10%) (Figure 2). When multiple possible diagnoses were provided by the referring clinician, the patient was classified as ‘Unclear’ (13%). The majority of the cohort were of indigenous African ancestry (~50%) (Figure 3).

### 3.2 NGS and Ion Torrent Software Analysis

All 135 patients were screened using the custom‐designed panel (Table 1). On average, 5,618,271 reads were obtained per sample with a mean depth of 2036X. Furthermore, 98.63% of the target bases had a minimum depth of 100X. ISP loading averaged 88%, with 64% usable reads and 29% polyclonality exhibited.

Investigation using the Ion AmpliSeq Designer Software (Thermo Fisher Scientific) revealed that none of the 27 amplicons identified to have dropped out in > 10% of the cohort were expected to exhibit uniform high coverage (Table S4).

The prior screening results from 25 patients, comprising 39 unique variants in 16 genes, were fully concordant with the variants called in the NGS data, thus demonstrating the sensitivity of the panel.

### 3.3 Combined Molecular Screening Results

**FIGURE 4:**
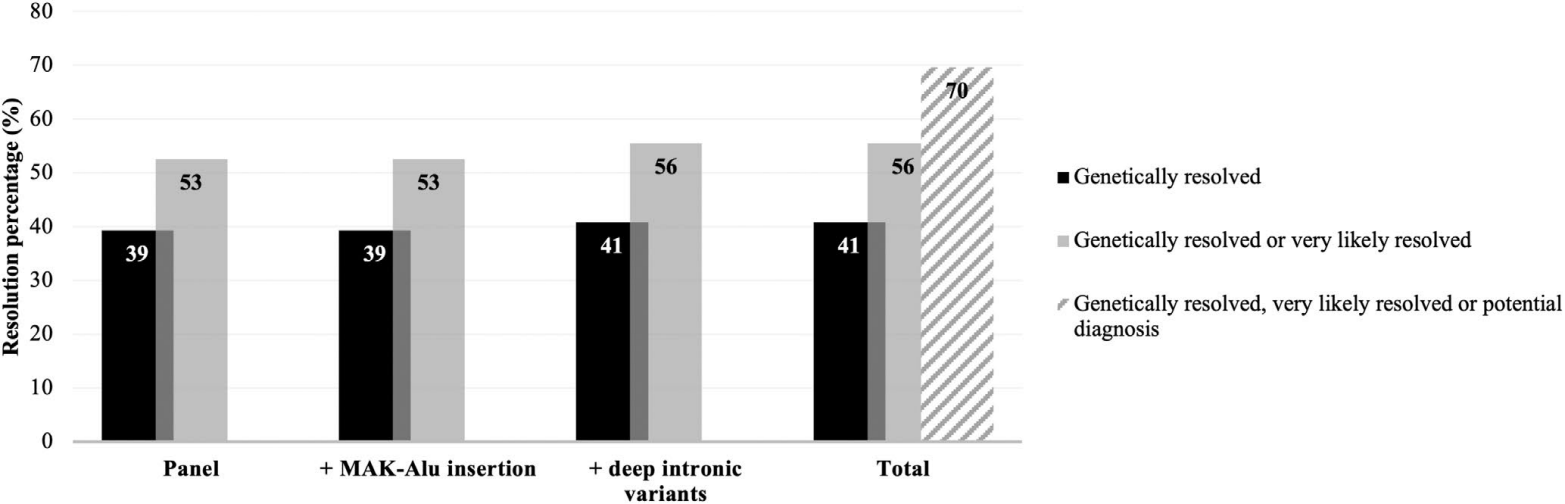
Resolution rate through the inclusion of a combined screening strategy.

Through the combined strategy of (1) NGS panel analysis, (2) *in silico MAK*‐Alu screening and (3) supplementary screening of seven functionally verified DIVs, a probable genetic diagnosis was obtained for 56% of the cohort. These patients were either classified as ‘genetically resolved’ or ‘very likely resolved’ (Figure 4). Nineteen unresolved patients were flagged with a ‘potential diagnosis’ as they carried either (i) a single heterozygous pathogenic/likely pathogenic variant and a heterozygous variant of unknown significance in an autosomal recessive gene, (ii) two heterozygous variants of unknown significance in an autosomal recessive gene or (iii) a single heterozygous variant of unknown significance in an autosomal dominant or X‐linked gene (Figure 4).

**FIGURE 5:**
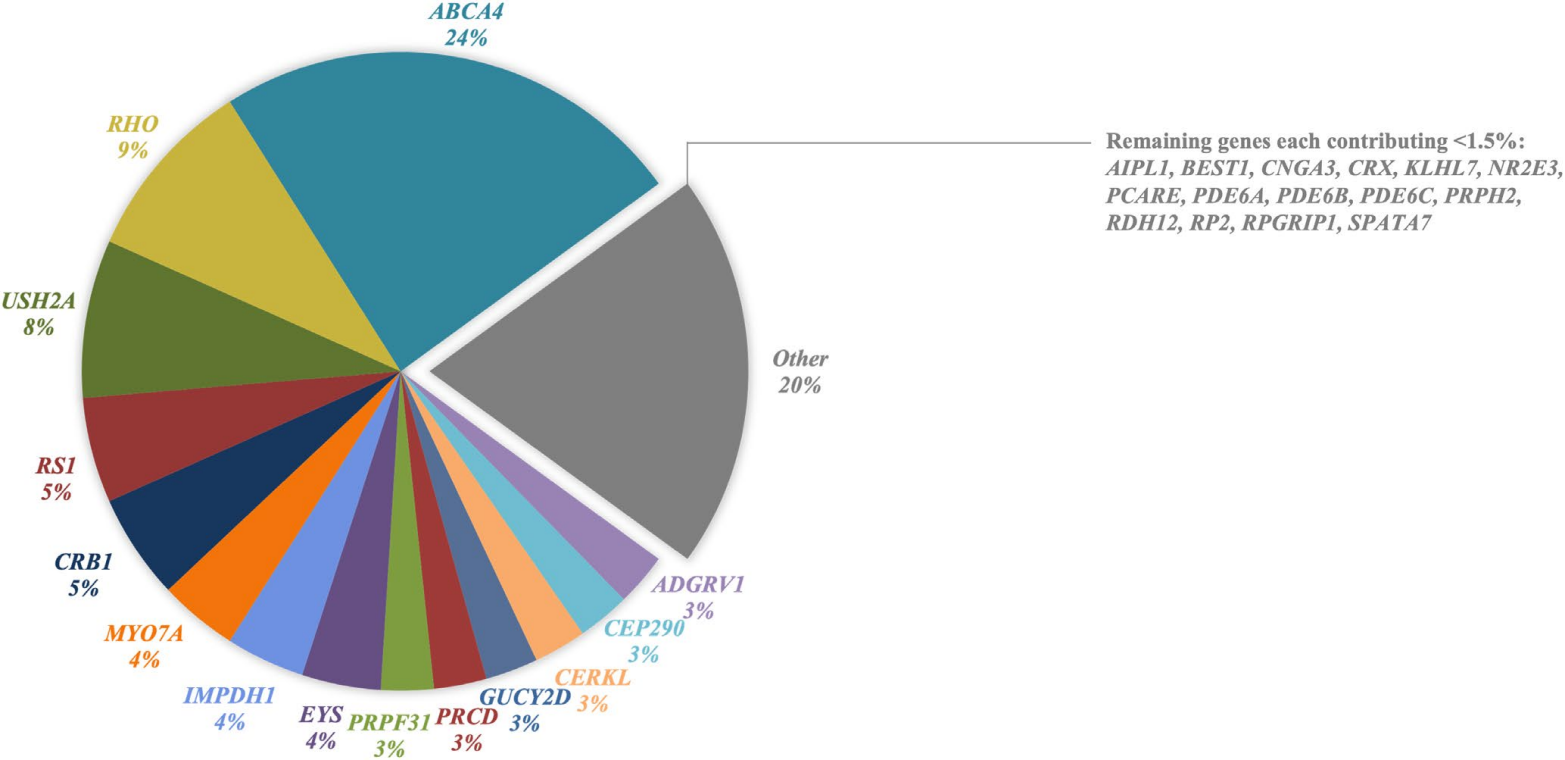
Breakdown of resolving genes.

The molecular diagnoses for all resolved patients (*n* = 75), along with the evidence of pathogenicity for each causal variant, are detailed in Table S5. Among the 464 unique variants interpreted according to the ACMG guidelines, 83 unique variants in 29 IRD genes were identified as causative for disease in the cohort (Figure 5).

**FIGURE 6:**
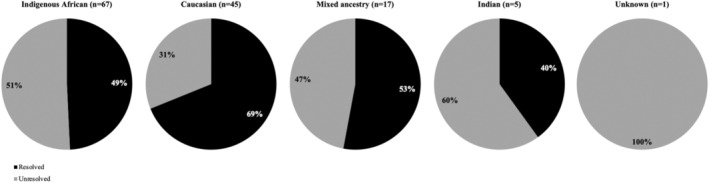
Percentage of probands with a probable genetic diagnosis per population group.

**FIGURE 7:**
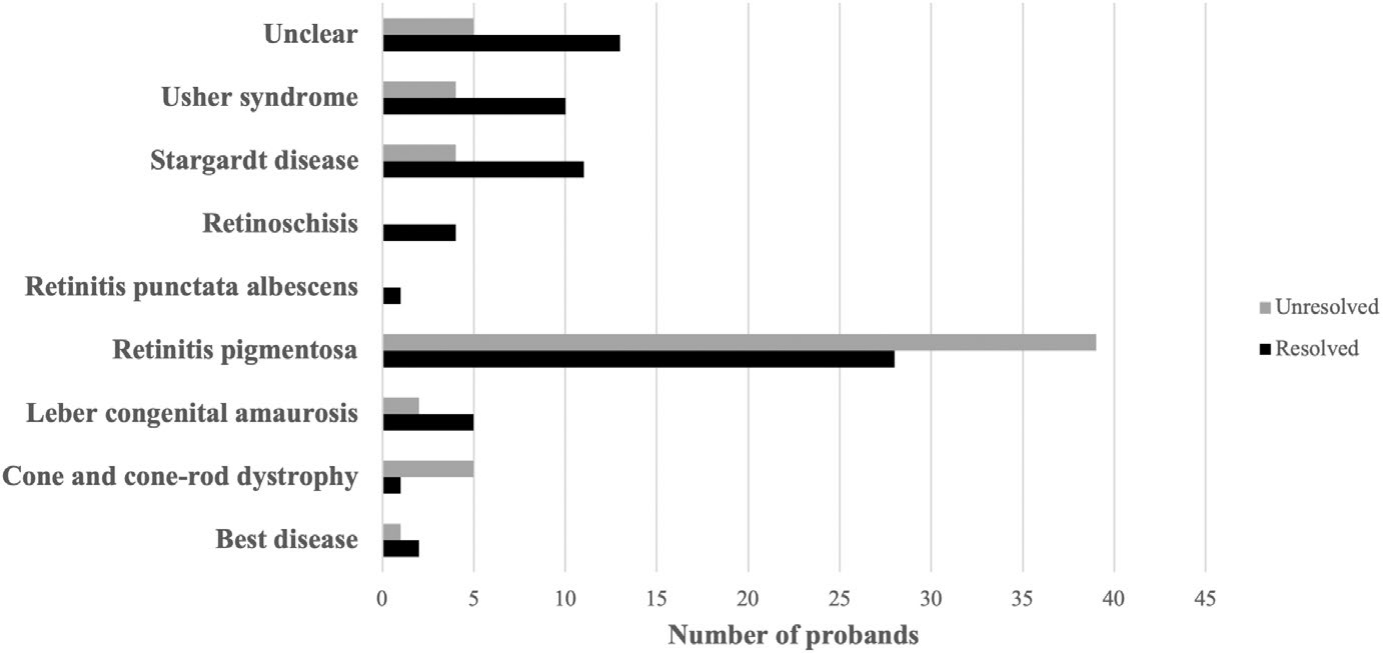
Number of probands with a probable genetic diagnosis per clinical classification.

**FIGURE 8:**
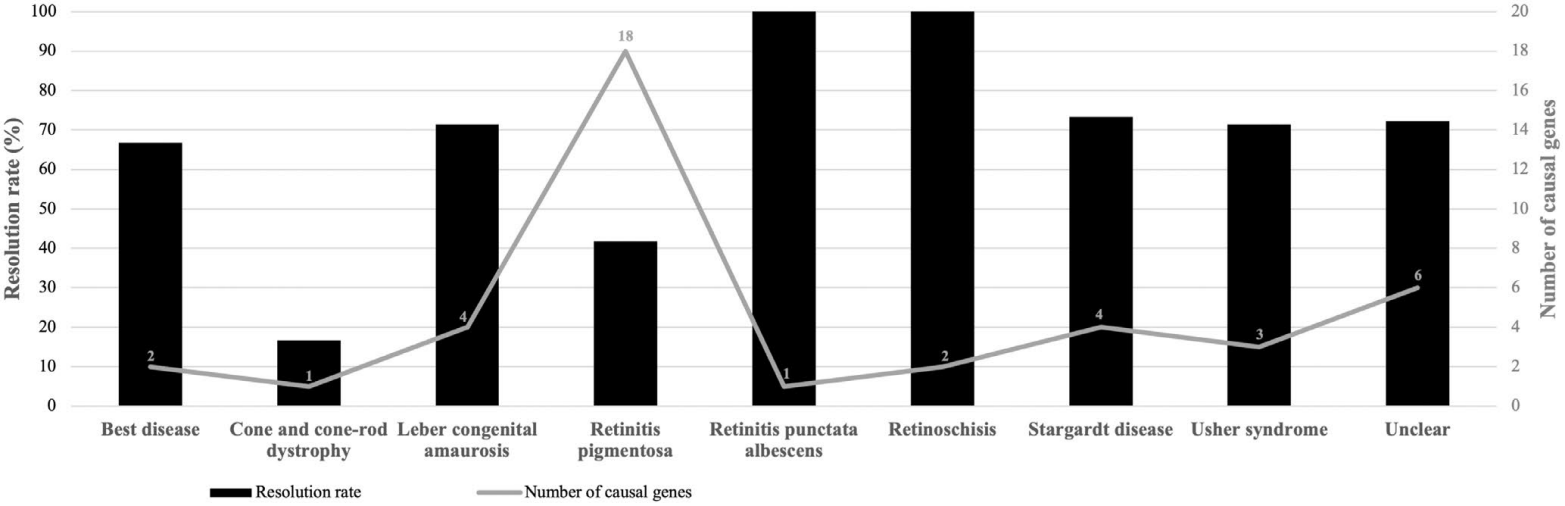
Resolution rates and number of identified causal genes per clinical classification.

Resolution rates differed among population groups, with the Caucasian group exhibiting the highest rate (69%) (Figure 6). Further variations in resolution rates across disorder categories, along with the corresponding number of identified causative genes, are illustrated in Figures 7 and 8.

Intriguingly, in ten patients, the molecular diagnosis did not align with the clinical diagnosis provided by the ophthalmologist (Table 4).

**TABLE 4:** Patients with discordant clinical and molecular diagnoses.

	Sex	Population group	Original clinical diagnosis	Age of onset (years)	Presumed Inheritance	Molecularly resolving gene and transcript	Associated disease and inheritance
Patient 13	M	C	RP	1	Recessive	*CEP290* NM_025114.4	arBBS, arLCA, ar syndromic/systemic diseases with retinopathy
Patient 17	M	C	RPA	4	Isolated	*RPGRIP1* NM_020366.4	arCD/CRD, arLCA, ar syndromic/systemic diseases with retinopathy
Patient 27	F	IA	RS	11	X‐Linked	*CRB1* NM_201253.3	arLCA, arRP, ad other retinopathy
Patient 54	F	IA	C/CRD	Not specified	Dominant	*IMPDH1* NM_000883.4	adLCA, adRP
Patient 57	M	IA	BEST	0.5	Recessive	*PDE6C* NM_006204.4	arCD/CRD
Patient 62	F	IA	STGD	18	Isolated	*BEST1* NM_001139443.2	arBEST, adMD, adRP, arRP, ad other retinopathy, ar other retinopathy
Patient 66	F	IA	Unclear: STGD or BEST	0	Isolated	*CNGA3* NM_001298.3	arCD/CRD, ar other retinopathy
Patient 67	F	IA	STGD	18	Isolated	*CERKL* NM_201548.5	arCD/CRD, arRP
Patient 69	M	IA	STGD	7	Recessive	*RS1* NM_000330.4	xl other retinopathy
Patient 74	F	IA	BEST	20	Unknown	*ABCA4* NM_000350.3	arCD/CRD, arMD, arRP, AMD

Abbreviations: ad, autosomal dominant; AMD, age‐related macular degeneration; ar, autosomal recessive; BBS, Bardet Biedl syndrome; BEST, Best disease; C, Caucasian; CD/CRD, cone or cone‐rod dystrophy; F, female; IA, Indigenous African; LCA, Leber Congenital Amaurosis; M, male; MD, macular degeneration; RP, retinitis pigmentosa; RPA, retinitis punctata albescens; RS, retinoschisis; STGD, Stargardt disease; xl, X‐linked.

Additionally, we identified 17 causal variants across nine genes, recurring in multiple patients of the cohort (Table 5).

**TABLE 5:** Causal variants identified in two or more patients of the cohort.

Gene and transcript	Coding	Protein	rsID	Number of probands		
				Indigenous African	Caucasian	Mixed ancestry
*ABCA4* NM_000350.3	c.1804C>T	p.Arg602Trp	rs61749409		4	1
*ABCA4* NM_000350.3	c.4634+1delG	p.(?)	No rsID	5		
*ABCA4* NM_000350.3	c.454C>T	p.(Arg152*)	rs62646861		2	1
*ABCA4* NM_000350.3	c.4469G>A	p.Cys1490Tyr	rs61751402		2	
*ABCA4* NM_000350.3	c.5603A>T	p.Asn1868Ile	rs1801466		3	
*ABCA4* NM_000350.3	c.4539+2001G>A	p.[=,Arg1514Leufs*36]	rs1457937638		2	
*ABCA4* NM_000350.3	c.6320G>A	p.Arg2107His	rs62642564	2		
*ABCA4* NM_000350.3	c.6617T>A^a^	p.(Leu2206His)	No rsID	2		
*CEP290* NM_025114.4	c.4723A>T	p.Lys1575*	rs137852834		1	1
*CERKL* NM_201548.5	c.365T>G	p.(Leu122Arg)	rs558913945	2		
*CRB1* NM_201253.3	c.2234C>T	p.(Thr745Met)	rs28939720	2		1
*EYS* NM_001142800.2	c.3443+1G>T	p.(?)	rs373441420	2		
*MYO7A* NM_000260.4	c.6377delC	p.(Pro2126Leufs*5)	No rsID	2		
*PRCD* NM_001077620.3	c.2T>C	p.(Met1?)	rs527236092	2		
*RHO* NM_000539.3	c.541G>A	p.Glu181Lys	rs775557680	1	1	
*USH2A* NM_206933.4	c.949C>A	p.=	rs111033272		3	
*USH2A* NM_206933.4	c.7595‐2144A>G	p.Phe287Tyrfs*69	rs786200928		2	

*Note:* () indicate predicted protein effect in accordance with the Human Genome Variation Society nomenclature. * indicate a translation termination (stop) codon, in accordance with Human Genome Variation Society nomenclature.

^a^ Putative novel variant.

We also uncovered 16 putative novel variants across 12 genes underlying disease in the cohort (Table 6). None of these variants have previously been reported in the literature or catalogued in ClinVar, to the best of our knowledge. Each novel variant was observed once in the cohort, with the exception of one *ABCA4* variant identified in two indigenous African probands. The ACMG classification and evidence of pathogenicity for each novel causative variant are listed in Table S6.

**TABLE 6:** Novel causal variants identified.

Chromosomal coordinates	Gene and transcript	Exon	Coding	Protein	Function
chr1:94548906	*ABCA4* NM_000350.3	7	c.858+2T>C	p.(?)	Splice
chr1:94463529	*ABCA4* NM_000350.3	48	c.6617T>A	p.(Leu2206His)	Missense
chr5:90041526	*ADGRV1* NM_032119.4	52	c.10888G>T	p.(Gly3630*)	Nonsense
chr5:89969877	*ADGRV1* NM_032119.4	23	c.4937_4938insCA	p.(Val1649Metfs*16)	Frameshift insertion
chr11:61723315	*BEST1* NM_001139443.2	3	c.193C>G	p.(Arg65Gly)	Missense
chr12:88457876	*CEP290* NM_025114.4	45	c.6152C>G	p.(Ser2051*)	Nonsense
chr1:197297897	*CRB1* NM_201253.3	2	c.416G>T	p.(Gly139Val)	Missense
chr17:7907173	*GUCY2D* NM_000180.4	3	c.725T>A	p.(Val242Glu)	Missense
chr17:7919272	*GUCY2D* NM_000180.4	17	c.3071C>T	p.(Thr1024Ile)	Missense
chr7:128038667	*IMPDH1* NM_000883.4	10	c.875G>T	p.(Gly292Val)	Splice
chr11:76858884	*MYO7A* NM_000260.4	4	c.174dup	p.(Met59Tyrfs*81)	Frameshift insertion
chr2:29293666	*PCARE* NM_001029883.3	1	c.3461delG	p.(Gly1154Alafs*18)	Frameshift deletion
chr3:129252546	*RHO* NM_000539.3	5	c.1032G>T	p.(Gln344His)	Missense
chrX:18660183	*RS1* NM_000330.4	6	c.616T>G	p.(Trp206Gly)	Missense
chr1:216420560	*USH2A* NM_206933.4	13	c.2176T>G	p.(Cys726Gly)	Missense
chr1:215848834	*USH2A* NM_206933.4	63	c.12419G>C	p.(Cys4140Ser)	Missense

*Note:* () indicate predicted protein effect in accordance with the Human Genome Variation Society nomenclature. * indicate a translation termination (stop) codon, in accordance with Human Genome Variation Society nomenclature.

## 4 Discussion

Targeted NGS panels are an invaluable tool for the molecular characterisation of IRD patients, particularly in resource‐limited settings, as they provide a focused, cost‐effective alternative to other NGS technologies whilst maintaining a high‐resolution rate. In this study, we employed a combined strategy to investigate 135 South African IRD patients. Our approach included NGS panel analysis, supplemented by *in silico MAK*‐Alu screening and testing for seven recurrent DIVs with an established functional impact.

Our approach successfully genetically resolved or very likely resolved 56% of the cohort, in line with other studies (Bravo‐Gil et al. 2016; Dockery et al. 2021; Neveling et al. 2012; Stone et al. 2017). The Caucasian subcohort exhibited the highest resolution rate (69%), likely due to the extensive genomic research conducted in this population and the high prevalence of *ABCA4* variants within the South African Afrikaner sub‐population (Roberts et al. 2012). In contrast, the indigenous African, mixed ancestry and Indian subcohorts yielded lower resolution rates (49%, 53% and 40%, respectively), likely attributed to the scarcity of variant information stemming from historical underrepresentation in genomics (Roberts 2017). However, our analysis demonstrated a substantial improvement for indigenous African patients. A prior screening strategy for South African IRD patients utilised Asper Ophthalmics microarrays, which were designed based on genetic findings predominantly from patients of European descent (Roberts et al. 2016). These microarrays resolved 41.2% of Caucasian patients but were less successful for indigenous African patients (12.8%) (Roberts et al. 2016). Our augmented NGS panel significantly improved resolution rates in both subcohorts, narrowing the diagnostic gap between these two population groups (Figure 6).

NGS panel analysis alone provided 53% of the cohort with a molecular diagnosis. Although the *in silico MAK*‐Alu screening did not enhance the resolution rate, it formed a valuable component of our investigation. No patients were found to harbour the *MAK*‐Alu insertion, which is unsurprising, as this variant has been identified as a founder mutation predominantly observed in the Jewish population (Venturini et al. 2015). The ethnoreligious background of our cohort was not available and the proportion of Jewish individuals in South Africa is relatively low (~0.2%) (Graham 2020), which likely contributed to the absence of this variant in our study. Nevertheless, this *in silico* analysis provided a worthwhile, cost‐free exploration of an IRD variant that was never before investigated in South Africa, contributing to the comprehensive nature of our approach and potentially facilitating the detection of similar variants in the future.

While the *MAK*‐Alu analysis did not improve the resolution rate, the supplementary screening of seven carefully selected DIVs successfully enhanced the overall resolution rate to 56% (Figure 4). DIVs can result in aberrant splicing by creating novel splice sites or altering splicing regulatory elements, which may induce exon skipping or pseudoexon inclusion (Cho et al. 2020; Vaz‐Drago, Custodio, and Carmo‐Fonseca 2017; Weisschuh, Buena‐Atienza, and Wissinger 2021). Although novel DIVs can be identified by WGS or full gene sequencing, the interpretation and classification of these variants are particularly challenging; *in silico* splicing prediction tools do not always accurately predict the effects on pre‐mRNA splicing (Sangermano et al. 2018), thus functional assays are essential to experimentally assess pathogenicity. Since the human retina is inaccessible and nonexpendable, in vitro minigene assays and multiple‐exon midigene assays have been developed (Sangermano et al. 2018). However, these methods do not provide the full genomic context or a retina‐specific model, which is crucial given the tissue‐specific nature of alternate splicing. Although patient‐derived induced pluripotent stem cells, differentiated into photoreceptor progenitor cells for functional analyses (Sangermano et al. 2016), offer a potential solution, this approach would be time‐consuming, costly and impractical to implement in a low‐resource setting. Therefore, we opted to supplement the panel by screening for a selection of published, functionally verified DIVs, providing a cost‐effective alternative that contributed to the increased resolution rate observed in this study.

In total, we identified 83 unique variants across 29 IRD genes underlying disease in the cohort (Figure 5), including 16 putative novel variants spanning 12 genes (Table 6). We adhered to the rigorous ACMG guidelines for variant classification; this is crucial as many variants historically reported as pathogenic are now known to have a high MAF or are observed in a homozygous state in healthy controls (Hanany and Sharon 2019; Lee and Garg 2015). Studies suggest that as many as 10% of reported pathogenic variants and 19% of genes previously associated with autosomal dominant IRDs are unlikely to be truly pathogenic (Hanany and Sharon 2019).

Seventeen causal variants across nine genes were identified in multiple patients within the cohort (Table 5). These comprised three of the seven *ABCA4* founder variants associated with STGD in the South African Caucasian population, particularly among individuals of Afrikaner descent (Roberts et al. 2012), as well as the common hypomorphic variant c.5603A>T in *ABCA4* (Midgley et al. 2020). Eight causal variants in seven genes appeared exclusively in multiple indigenous African patients, including the founder *MYO7A* c.6377delC variant (Roberts et al. 2015) and the frequent *CERKL* c.365T>G variant previously reported in this population (Roberts et al. 2016). Of particular interest was the recurrent splice variant c.4634+1delG in *ABCA4*, detected in five indigenous African patients, all diagnosed with MD/STGD. This variant was first reported in three indigenous African patients in the landmark *ABCA4* deep sequencing study (Khan et al. 2020), and to the best of our knowledge, has not been reported elsewhere. Our custom panel thus detected variants known to be common contributors to the local IRD disease burden and identified additional variants that may influence the genetic landscape of IRDs in South Africa.

Further investigation into the causative variants revealed that 14 genes accounted for approximately 80% of the resolved IRD patients in our cohort. This is consistent with other reports; in a recent study in the United Kingdom, 70% of resolved patients were attributed to variants in 20 genes, while in Canada and the United States, 70% of resolved patients were attributed to variants in only 10 genes (Dockery et al. 2021; Goetz et al. 2020; Pontikos et al. 2020). Consistent with our findings, these studies also identified *ABCA4* and *USH2A* as major contributors to the overall IRD disease burden. The high detection rates observed in *ABCA4* and *USH2A* may be influenced by the clinical classification of the cohort and gene size; *ABCA4* and *USH2A* are among the largest genes included in our panel design. The other major contributing genes generally depend on the relative proportion of each specific IRD included. For example, *RHO* variants are the most common cause of autosomal dominant RP (Zhen et al. 2023); seven patients from our cohort (three Caucasian and four indigenous African), all clinically diagnosed with autosomal dominant RP, were genetically resolved with six unique *RHO* variants (Table S5). Consequently, *RHO* was the second most frequently mutated gene in this study (9%), likely attributable to the comparatively higher proportion of autosomal dominant RP patients within the cohort (Figure 6) (Table S5).

Underscoring the importance of these findings, numerous clinical trials for IRD gene therapy are ongoing, where patient eligibility for inclusion requires knowledge of the disease‐causing gene, and in some cases, the exact causal variant(s). Of the 29 causative genes identified in this study, 14 are currently the basis of a clinical trial (https://clinicaltrials.gov/).

RP patients constituted the majority of our cohort (50%) (Figure 2) and RP emerged as the most genetically heterogeneous disorder, with causal variants identified in 18 genes. This substantial heterogeneity may partially explain the higher number of unresolved RP patients observed in our study (Figures 7 and 8). Interestingly, the ‘Unclear’ clinical category, which comprised 13% of the cohort, exhibited a higher resolution rate (72%) than expected. Although patients in this category received a COD from an ophthalmologist, each patient had multiple possible clinical diagnoses due to the inherent complexity of IRDs. The molecular diagnosis successfully refined/assigned a clinical diagnosis for three of these patients, demonstrating the utility of the employed analysis pipeline. We utilised a hybrid strategy that included phenotype‐first variant filtration, during which we selectively investigated disease‐associated genes, followed by a gene‐agnostic approach for unresolved samples that expanded the investigation to all genes included in the panel. This allowed us to limit our FGR whilst negating the pitfalls of phenotype‐specific panels. Although utilising highly specific phenotype‐based panels offers numerous advantages, caution must be exercised; in a recent study by Dillion et al. (2018), 23% of paediatric patients with various monogenic disorders would not have been molecularly diagnosed if they were screened with the proposed phenotype‐specific panel (Dillon et al. 2018; Dockery et al. 2021).

On this note, the molecular diagnosis of ten patients did not align with the clinical diagnosis provided by the ophthalmologist (Table 4), and we therefore recommend these patients should undergo clinical re‐examination. This discrepancy, particularly prevalent within the indigenous African subcohort, underscores the potential influence of modifier variants on phenotypic manifestations.

Unexpected molecular findings emerged even among the ‘simpler’ monogenic IRDs in our study. Three of the four patients clinically diagnosed with X‐linked RS harboured causative *RS1* variants, as expected. However, the remaining patient (Patient 27 in Table 4, an indigenous African) did not carry a likely causal *RS1* variant but instead harboured two pathogenic *CRB1* variants. X‐linked RS is clinically characterised by splitting of the macula and peripheral retina, whereas mutations in the *CRB1* gene are usually associated with severe retinal dystrophies exhibiting variable phenotypes. Upon closer inspection of the literature, biallelic *CRB1* variants have been previously associated with foveal retinoschisis, specifically characterised by splitting in the foveal region of the retina (Vincent et al. 2016). In contrast, Patient 69 in Table 4 (also an indigenous African) was clinically diagnosed with autosomal recessive STGD, a form of macular degeneration primarily associated with the *ABCA4* gene, but did not carry any likely causal *ABCA4* variants. Instead, a known, pathogenic variant was identified in *RS1*, which is associated with RS and is phenotypically distinct from STGD (Sauer et al. 1997). The distinct phenotypes observed in indigenous African patients suggest potential modifier variants or the influence of a markedly different environmental and epigenetic landscape within this genetically diverse cohort, setting them apart from other reported IRD cases (Roberts et al. 2016).

This study facilitated the compilation of a comprehensive compendium of 105 Ion Torrent artefacts occurring within IRD genes. This may benefit the international community, as five variants flagged as artefacts are listed as pathogenic/likely pathogenic in ClinVar. For example, the *PROM1* c.1696delA variant has one submission in ClinVar as likely pathogenic from 2016; however, through interrogation in IGV, this 1 bp deletion appeared in every sample of this cohort and is thus likely an artefact. This finding aligns with a similar report by Lumaka et al. (2022), where 41 variants within the ClinVar database were identified in more than 5% of the H3Africa participants, allowing these variants to be subsequently reclassified as benign (Lumaka et al. 2022). This underscores the importance of continually scrutinising and updating variant databases for improved accuracy in variant interpretation.

Twelve unresolved patients in this cohort carried a single pathogenic/likely pathogenic variant in an autosomal recessive gene. Even following the investigation of all variants in the relevant genes, no additional likely disease‐causing variants were identified. Mono‐allelic variants are a well‐known occurrence in IRDs; the corresponding second allele may harbour an elusive class of variant, or the initial variant may simply be coincidental, as it is estimated that 36% of the global population are healthy carriers of a single pathogenic/likely pathogenic variant in an autosomal IRD gene (Hanany, Rivolta, and Sharon 2020). This supports the rationale underlying our analysis pipeline, in which we initially restricted variant investigation to minimise the FGR (Stone et al. 2017).

The unresolved patients in this study may carry causative variants in genes not included in the panel (or in the panel version utilised), or in regions not sequenced, such as the *RPGR* ORF‐15 mutation hotspot (Maggi et al. 2020) or intronic regions. Additionally, these patients may harbour causative structural variants, which were not investigated in this study. Structural variants, including large duplications, inversions and translocations are not readily detected by targeted NGS due to the use of short read lengths that cover select regions of the genome, typically focusing only on exonic sequences, thereby limiting the ability to accurately reconstruct and identify their full genomic context.

This study encountered several limitations that primarily impacted the interpretation and classification of variants. First, the lack of comprehensive clinical information for each patient made it challenging to correlate genetic findings with clinical phenotype, potentially affecting the precision of variant classification. Second, the unavailability of familial DNA samples impeded our ability to perform co‐segregation analysis, which is crucial for confirming the inheritance pattern and the segregation of variants with the disease, as well as for identifying the phase of variants in recessive genes. Third, the significant scarcity of information on variants identified in indigenous African patients hampered our ability to accurately classify these variants. Consequently, six of the nine patients flagged with a potential molecular diagnosis were from the indigenous African subcohort.

Therefore, we recommend ensuring the availability of familial DNA samples for co‐segregation analysis and obtaining comprehensive clinical information whenever possible. Additionally, as all causal variants identified in the NGS data were successfully verified by Sanger sequencing, routine Sanger sequencing verification is not deemed necessary; instead, we recommend thorough variant interrogation using IGV. This echoes a similar finding by Beck et al. (2016), which advises against routine Sanger sequencing validation of NGS variants, noting a high validation rate of at least 99.965% for NGS data across a large dataset.

Our study demonstrates that the targeted NGS panel is a cost‐effective approach with robust diagnostic performance. While WGS and WES provide more comprehensive data, this introduces additional data complexity, incidental findings and increased storage costs. In contrast, the targeted NGS panel delivers customisable, focused results, achieving an optimal balance between diagnostic yield and cost‐efficiency, outperforming both broader NGS technologies and traditional microarrays. The 56% resolution rate achieved in our cohort underscores the clinical utility of the NGS panel, providing patients with essential molecular diagnoses that enable genetic counselling, aid in patient management and family planning and facilitate access to gene‐based clinical trials, ensuring more precise management of IRDs.

## 5 Conclusion

This marks the first report of a custom IRD panel tailored specifically for use in Africa as a first‐tier screening tool for IRDs. This augmented panel demonstrates strong diagnostic performance, achieving a 56% resolution rate in line with international studies, making it a valuable tool for IRD diagnostics in resource‐limited settings. Our findings underscore the importance of continued efforts to expand genomic databases and improve variant classification accuracy in diverse populations.

## Author Contributions

Nicole Midgley collected the data, contributed to data and analysis tools, performed the analysis and wrote and edited the manuscript. Lisa Roberts conceived and designed the analysis and supervised the project. George Rebello assisted with project supervision and analysis tools. Lara K. Holtes assisted with data collection. Raj Ramesar assisted with project supervision and provided resources. All authors contributed to editing the manuscript.

## Conflicts of Interest

The authors declare no conflicts of interest.

## Supporting information


Data S1.

## Data Availability

The data that supports the findings of this study are available in the Supporting Information of this article.
